# A comparison of trends in stroke care and outcomes between in-hospital and community-onset stroke – The South London Stroke Register

**DOI:** 10.1371/journal.pone.0212396

**Published:** 2019-02-21

**Authors:** Eva S. Emmett, Abdel Douiri, Iain J. Marshall, Charles D. A. Wolfe, Anthony G. Rudd, Ajay Bhalla

**Affiliations:** 1 School of Population Health & Environmental Sciences, King’s College London, London, United Kingdom; 2 NIHR Comprehensive Biomedical Research Centre, Guy’s and St Thomas’ NHS Foundation Trust and King’s College London, London, United Kingdom; 3 NIHR Collaboration for Leadership in Applied Health Research and Care, Guy’s and St Thomas’ NHS Foundation Trust and King’s College London, London, United Kingdom; 4 Department of Ageing and Health, Guy’s and St Thomas’ NHS Foundation Trust, London, United Kingdom; Dartmouth-Hitchcock Medical Center, UNITED STATES

## Abstract

**Background:**

Stroke care and outcomes have improved significantly over the past decades. It is unclear if patients who had a stroke in hospital (in-hospital stroke, IHS) experienced similar improvements to those who were admitted with stroke (community-onset stroke, COS).

**Methods:**

Data from the South London Stroke Register were analysed to estimate trends in processes of care and outcomes across three cohorts (1995–2001, 2002–2008, 2009–2015). Kaplan-Meier survival curves were calculated for each cohort. Associations between patient location at stroke onset, processes of care, and outcomes were investigated using multiple logistic regression and Cox proportional hazards models.

**Results:**

Of 5,119 patients admitted to hospital and registered between 1995 and 2015, 552(10.8%) had IHS. Brain imaging rates increased from 92.4%(COS) and 78.3%(IHS) in 1995–2001 to 100% for COS and IHS in 2009–2015. Rates of stroke unit admission rose but remained lower for IHS (1995–2001: 32.2%(COS) vs. 12.4%(IHS), 2002–2008: 77.1%(COS) vs. 50.0%(IHS), 2009–2015: 86.3%(COS) vs. 65.4%(IHS)). After adjusting for patient characteristics and case-mix, IHS was independently associated with lower rates of stroke unit admission in each cohort (1995–2001: OR 0.49, 95%CI 0.29–0.82, 2002–2008: 0.29, 0.18–0.45, 2009–2015: 0.22, 0.11–0.43). In 2009–2015, thrombolysis rates were lower for ischaemic IHS (17.8%(COS) vs. 13.8%(IHS)). Despite a decline, in-hospital mortality remained significantly higher after IHS in 2009–2015 (13.7%(COS) vs. 26.7%(IHS)). Five-year mortality rates declined for COS from 58.9%(1995–2001) to 35.2%(2009–2015) and for IHS from 80.8%(1995–2001) to 51.1%(2009–2015). In multivariable analysis, IHS was associated with higher mortality over five years post-stroke in each cohort (1995–2001: HR 1.27, 95%CI 1.03–1.57, 2002–2008: 1.24, 0.99–1.55, 2009–2016: 1.39, 0.95–2.04).

**Conclusions:**

Despite significant improvements for IHS patients similar to those for COS patients, rates of stroke unit admission and thrombolysis remain lower, and short- and long-term outcomes poorer after IHS. Factors preventing IHS patients from entering evidence-based stroke-specific hospital pathways in a timely fashion need further investigation.

## Introduction

Between 4% and 17% of all strokes occur in patients while in hospital[1, 2]. Although patients with in-hospital stroke (IHS) avoid any pre-hospital delays and could potentially be diagnosed and treated rapidly, they represent a particular challenge: pre-existing medical conditions can mimic or obscure stroke symptoms and delay or prevent diagnosis[3]. Co-morbidities, e.g. major surgery or other life-threatening acute conditions, can be contra-indications to acute treatments such as thrombolysis[4]. IHS are generally more severe, leading, together with the initial admission diagnosis, to longer hospital stays, higher in-hospital mortality, and worse functional outcomes at discharge[2, 5–8].

In addition to these intrinsic challenges, a “quality gap” has been found in the care of IHS compared to community-onset stroke (COS), with lower proportions of eligible IHS patients receiving deficit-free, i.e. fully guideline-adherent care[2]. Delays to diagnosis and treatment, and lower rates of brain imaging, stroke unit (SU) admission, thrombolysis for eligible IHS patients, and secondary prevention have been widely observed, including in a previous study from the South London Stroke Register[2–5, 8, 9].

Several studies in the last 10 years have shown that short- and long-term outcomes after stroke generally have improved over the last few decades[10, 11], thought to be associated with better acute care, such as thrombolysis[12] and SU care[13], as well as risk factor management before and after stroke[10]. However, studies investigating how care and outcomes of IHS have changed over time are lacking. It is therefore unclear if IHS patients have benefitted equally from the recent improvements in stroke care and outcomes or whether the reported gap between IHS and COS has worsened or improved. Additionally, no previous studies have investigated functional outcome after IHS beyond three months or mortality beyond one year after stroke.

The aim of this study was to compare temporal trends of acute care and short- and long-term outcomes of IHS and COS using data from the South London Stroke Register (SLSR), an ongoing inner-city register established in 1995.

## Methods

### Identification of patients

The SLSR is a population-based register, continuously ongoing since January 1995, recording all first-in-a-lifetime strokes in a geographically defined, inner-city area of South London. At the 2001 UK Census, the area comprised a multi-ethnic population of 310,026 people (63% white, 28% black, 9% other), while at the 2011 Census the population had increased to 357,308 people (56% white, 25% black, 18% other).

Multiple overlapping sources of notification were used by trained study nurses and fieldworkers to maximise case ascertainment. These sources included hospital wards, neurovascular outpatient clinics, radiology requests, A&E records, and general practitioners. Capture-recapture models estimated completeness of case ascertainment to be ~88%[14]. Stroke diagnosis was verified by a study clinician, using the World Health Organization clinical definition[15].

### Ethical approval

The study and its consent procedure were approved by the ethics committees of Guy’s and St Thomas’ Hospital Trust, King’s College Hospital, Queen’s Square, and Westminster Hospital.

All patients, or their next of kin if the patient did not have capacity to consent (e.g. due to cognitive impairment, decreased consciousness, or expressive/receptive dysphasia), gave written informed consent to participate in the study. The fieldworker approaching the patient was responsible for determining capacity to consent. Few patients declined to be registered (1%)[16].

### Socio-demography, pre-stroke vascular risk factors, and case mix

Sociodemographic data collected included age, sex, ethnic origin (self-defined using the 1991 Census question stratified into white, black, and other ethnic background) and socioeconomic status (manual or non-manual occupation according to the patient’s current or most recent employment).

Pre-stroke diagnoses of vascular risk factors were ascertained from general practitioners or hospital records and included hypertension, hypercholesterolaemia, atrial fibrillation (AF), transient ischaemic attack (TIA), ischaemic heart disease, diabetes mellitus, and smoking (ex-smoker or current).

As the National Institutes of Health Stroke Scale (NIHSS)[17] was introduced into the SLSR as a measure of stroke severity in 2004, it could not be used for the analysis of trends since 1995. Instead, case mix was analysed using the Glasgow Coma Scale (GCS) score (dichotomized: <13/≥13)[18], dysphagia (3-oz water swallow test), and incontinence at time of maximum impairment. Additionally, the Barthel Index (BI)[19] was recorded as a measure of disability pre-stroke and at 7 days post-stroke (or at discharge if earlier). A BI score of <15 was used to identify patients with moderate (BI = 10–14) or severe (BI<10) disability[20].

Stroke subtype was classified according to the Oxford Community Stroke Project classification[21].

### Processes of care

Brain imaging (stratified into no imaging, any imaging (CT and/or MRI), MRI), swallowing assessment, SU admission (at any point during hospital stay), >50% of post-stroke hospital stay spent in a SU, and thrombolysis rates (denominator: ischaemic strokes) were analysed as measures of acute hospital care. Length of hospital stay was calculated as length of hospital admission for COS or time between stroke onset and discharge for IHS.

Discharge destination was stratified into home without carer, home with carer or sheltered accommodation, and nursing care.

At three months post-stroke, data on rehabilitation therapies (physiotherapy (PT), occupational therapy (OT), and speech and language therapy (SALT)) at any time since discharge were analysed. Rates of PT or OT were based on those eligible, i.e. those with motor, sensory, or visual deficit in the acute phase, while rates of SALT were based on those with dysphasia or dysarthria in the acute phase. Prescribed secondary prevention therapy at three months post-stroke included antihypertensive and statin therapy in those with a diagnosis of hypertension and hypercholesterolaemia respectively. Rates of antiplatelet therapy were based on those with ischaemic stroke, and anticoagulant therapy on those with ischaemic stroke and AF.

### Outcome measures

Follow-up assessments were performed at three months, one year, and then annually by face-to-face interview or postal questionnaire. Follow-up data were censored on 30 November 2017. At all follow-up points, activities of daily living were recorded using the BI. Date of death was ascertained from hospital records, general practitioners, and the Office for National Statistics.

### Statistical methods

Patients were stratified into community-onset stroke (COS) or in-hospital stroke (IHS). Both groups were further stratified into three consecutive seven-year cohorts according to year of stroke (1995–2001, 2002–2008, 2009–2015).

Unadjusted differences between COS and IHS were investigated using Chi-squared test for binary variables, the unpaired t-test for age, and the Mann-Whitney U test for length of hospital admission. These analyses were repeated for each cohort. *P* values for trend were calculated for both patient groups using the Cochran-Armitage tests for trend. Multivariable logistic regression was used to calculate adjusted estimates of the impact of patient location at stroke (COS or IHS) on receipt of appropriate care in the acute phase, in-hospital death, and discharge destination for each cohort. Adjustment variables were socio-demographic factors (age, sex, ethnicity, and socioeconomic status), pre-stroke vascular risk factors, case mix variables (GCS score, incontinence, dysphagia, and BI), stoke subtype, and SU admission (for discharge destination).

**Fig 1:**
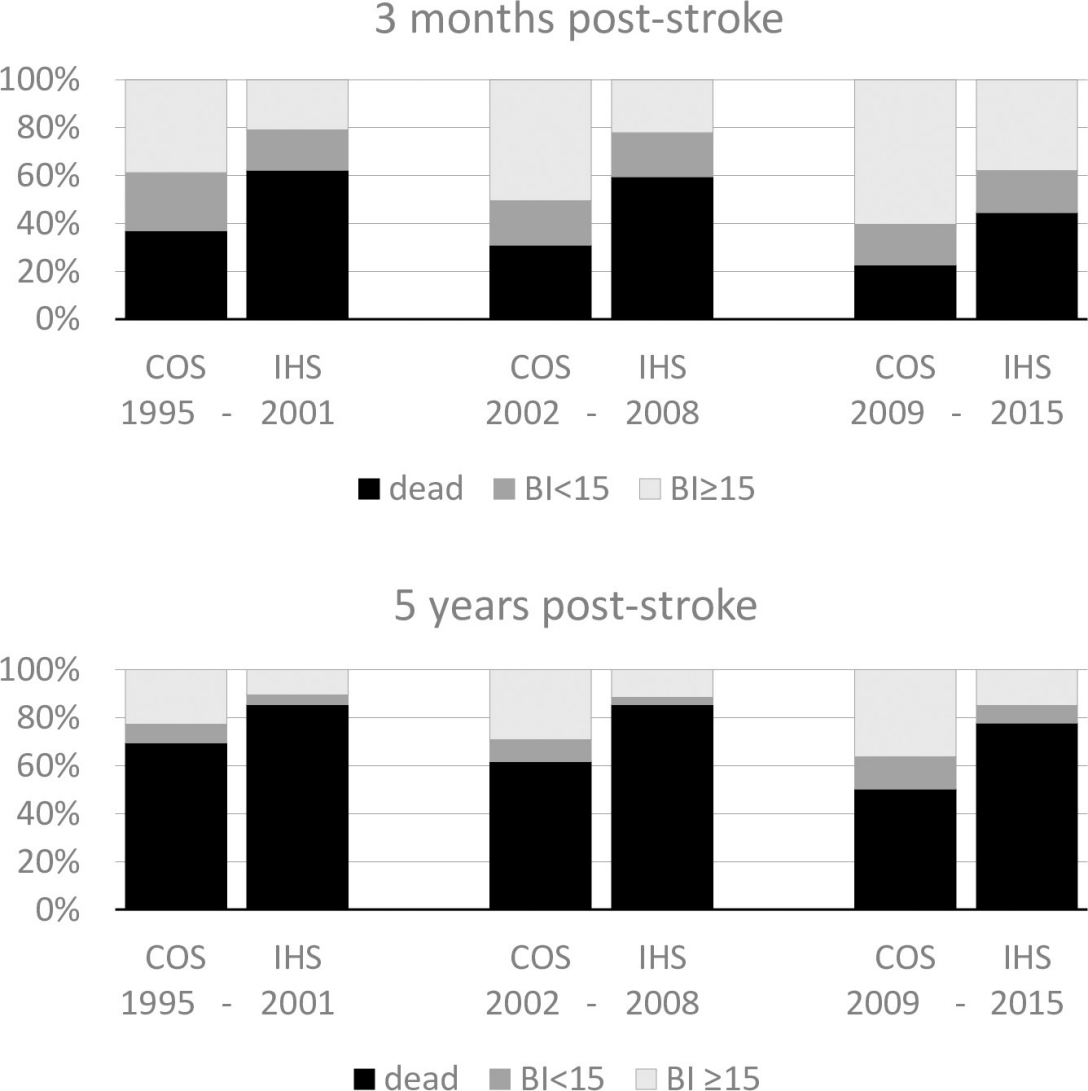
Proportion of dead, severely/moderately disabled (BI<15), or mildly/not disabled (BI≥15) patients. Abbreviations: BI Barthel Index.

Three-month and five-year outcomes were stratified into dead, moderately/severely disabled (BI<15), and mildly disabled/independent (BI≥15). Proportions of five-year outcomes (Fig 1) refer to patients who have reached five years post-stroke.

Survival analyses up to five years after COS and IHS were performed stratified by cohort using the Kaplan-Meier method (unadjusted) and Log-rank tests. For each cohort, multivariate survival analysis was undertaken using cox proportional hazards models to estimate the prognostic value of IHS on survival, adjusting for socio-demographic factors, pre-stroke vascular risk factors, case mix, stroke subtype, SU admission, and imaging.

To assess the robustness of the results to missing data, we performed sensitivity analyses using multiple imputation of missing data based on inverse probability weights. The probability of response was estimated with multivariable logistic regression including factors associated with dropping out (cognitive score at previous visits, age, socioeconomic status, and ethnicity). This approach had little effect on the estimates. The observed data analysis was therefore used for the present study.

All tests were 2 tailed, and *P* values <0.05 were considered statistically significant. All statistical analyses were performed using Stata 14.2 (StataCorp, Texas, USA).

## Results

5,790 stroke patients were registered between January 1995 and December 2015. 604 (10.4%) non-hospitalised patients were excluded from the analysis, as well as 67 (1.2%) patients with unknown patient location at the time of stroke onset. Out of 5119 admitted stroke patients, 552 (10.8%) experienced a stroke while admitted to hospital for another cause, with the proportion declining from 12.4% in 1995–2001 to 10.3% in 2002–2008, and 9.5% in 2009–2015.

50.6% of IHS patients experienced a stroke on an acute medical ward (managing various conditions relating to cardiology, endocrinology, or gastroenterology, and respiratory or infectious diseases), whereas the remainder were on surgical/neurosurgical wards (15.7%/0.9%), ITU/HDU (13.6%), geriatric wards (9.1%), or other wards (9.4%).

Table 1 illustrates changes in baseline characteristics over time. Average age of COS and IHS patients at index stroke fell and the significant age difference between COS and IHS seen in the earlier cohorts (P<0.001) was not significant in 2009–2015 (P = 0.150). The proportion of white ethnicity declined in both groups, mirroring the trend in the source population, with a significantly larger proportion of white ethnicity in the IHS compared to the COS group continuing across all three cohorts. While the pre-stroke diagnosis of ischaemic heart disease declined in both groups, it was significantly more common in IHS patients in each cohort (P<0.001). IHS patients continued to have more pre-stroke disability and more clinical impairments after stroke than COS patients, despite an improvement in impairments. There was a significant decline of the proportion of total anterior circulation infarcts in COS and IHS patients.

**Table 1:** Baseline characteristics of the study population by cohort.

	COS			IHS			*P for trend*	
	1995–2001	2002–2008	2009–2015	1995–2001	2002–2008	2009–2015	*COS*	*IHS*
**Cohort (%)**	1,512(87.7)	1,792(89.7)	1,263(90.5)	213 (12.4)	206 (10.3)	133 (9.5)	*0*.*010*	*0*.*010*
**Age**, mean (SD)	70.6 (14.5)	69.2 (15.5)	67.6 (16.2)	74.2 (13.3)	74.4 (12.7)	69.8 (16.4)	*<0*.*001*	*0*.*024*
**Sex**, male (%)	746 (49.3)	920 (51.3)	666 (52.7)	85 (39.9)	104 (50.5)	76 (57.1)	*0*.*073*	*0*.*001*
**Ethnicity**								
White (%)	1,125(75.5)	1,190(68.0)	713 (57.8)	175 (85.0)	154 (77.8)	86 (67.2)	*<0*.*001*	*<0*.*001*
Black (%)	288 (19.3)	440 (25.2)	428 (34.7)	29 (14.1)	29 (14.7)	29 (22.7)	*<0*.*001*	*0*.*005*
**Socioeconomic status**								
Manual (%)	854 (69.3)	938 (64.0)	399 (52.9)	95 (66.4)	101 (64.3)	34 (48.6)	*<0*.*001*	*0*.*022*
**Pre-stroke risk factors**								
Hypertension	866 (62.1)	1,160(65.7)	796 (64.5)	133 (65.2)	144 (70.2)	89 (68.5)	*0*.*183*	*0*.*457*
Hypercholesterolaemia	59 (9.9)	393 (22.4)	461 (37.9)	7 (10.3)	58 (28.6)	56 (43.4)	*<0*.*001*	*<0*.*001*
Atrial fibrillation	268 (19.0)	237 (13.4)	233 (19.1)	58 (28.9)	54 (26.5)	33 (26.4)	*0*.*893*	*0*.*595*
Ischaemic heart disease	275 (19.5)	267 (15.3)	123 (10.1)	68 (33.2)	66 (32.7)	26 (20.8)	*<0*.*001*	*0*.*028*
Diabetes mellitus	245 (17.3)	339 (19.2)	288 (23.3)	33 (15.9)	58 (28.6)	42 (32.6)	*<0*.*001*	*<0*.*001*
Smoking	353 (38.2)	528 (46.5)	326 (39.2)	57 (44.9)	70 (54.7)	44 (51.8)	*0*.*594*	*0*.*258*
**Case mix**								
Pre-stroke BI <15	113 (6.3)	102 (5.5)	103 (8.3)	32 (15.8)	43 (23.1)	18 (15.8)	*0*.*051*	*0*.*732*
GCS <13	438 (29.4)	479 (27.5)	296 (24.2)	96 (47.8)	98 (51.9)	32 (29.1)	*0*.*003*	*0*.*007*
Incontinence	741 (51.7)	790 (45.7)	358 (29.7)	144 (74.2)	140 (71.8)	58 (49.6)	*<0*.*001*	*<0*.*001*
Dysphagia	688 (48.7)	586 (36.7)	245 (22.9)	134 (66.3)	109 (61.9)	30 (30.9)	*<0*.*001*	*<0*.*001*
NIHSS >14	*	333 (27.0)	182 (17.7)	*	65 (44.2)	26 (27.1)	*n/a*	*n/a*
Post-stroke BI (7d) <15	735 (69.0)	872 (57.0)	423 (39.4)	122 (83.0)	130 (79.8)	54 (54.6)	*<0*.*001*	*<0*.*001*
**Stroke subtype**								
TACI	260 (18.4)	214 (12.5)	145 (13.2)	51 (28.3)	36 (19.3)	15 (14.4)	*<0*.*001*	*0*.*004*
PACI	329 (23.2)	503 (29.3)	370 (33.7)	39 (21.7)	71 (38.0)	42 (40.4)	*<0*.*001*	*<0*.*001*
POCI	154 (10.9)	208 (12.1)	138 (12.6)	21 (11.7)	24 (12.8)	17 (16.4)	*0*.*178*	*0*.*281*
LACI	335 (23.7)	447 (26.0)	256 (23.3)	37 (20.6)	41 (21.9)	9 (8.7)	*0*.*938*	*0*.*029*
PICH	238 (16.8)	251 (14.6)	150 (13.7)	25 (13.9)	12 (6.4)	14 (13.5)	*0*.*026*	*0*.*589*
SAH	100 (7.1)	94 (5.5)	39 (3.6)	7 (3.9)	3 (1.6)	7 (6.7)	*<0*.*001*	*0*.*372*

Data are count (%, excluding those with missing values) unless otherwise indicated

*not collected during that time period

Abbreviations: BI Barthel Index, GCS Glasgow Coma Scale, NIHSS National Institutes of Health Stroke Scale, TACI total anterior circulation infarct, PACI partial anterior circulation infarct, POCI posterior circulation infarct, LACI lacunar infarct, PICH primary intracerebral haemorrhage, SAH subarachnoid haemorrhage

Table 2 shows rates of care interventions in the acute and early phase post-stroke stratified by patient location and cohort. While IHS patients were less likely than COS patients to have brain imaging in 1995–2001 (COS:92.4% vs. IHS 78.3%, P<0.001), imaging frequency increased to 100% in both groups in 2009–2015. In contrast, the frequency of swallowing assessments declined from 94.4% (COS) and 95.3% (IHS) in 1995–2001 (P = 0.594) to 84.9% (COS) and 72.9% (IHS) in 2009–2015 (P<0.001). SU admission rose significantly in both groups from 32.2% and 12.4% in 1995–2001 (P<0.001) to 86.3% and 65.4% in 2009–2015 (P<0.001) for COS and IHS respectively. Similarly, the proportion of patients spending over 50% of their hospital admission on a SU increased significantly in both patient groups. Thrombolysis rates were 10.0% (COS) and 5.1% (IHS) in 2002–2008 (P = 0.064), compared to 17.8% (COS) and 13.8% (IHS) in 2009–2015 (P = 0.290).

**Table 2:** Rates of care interventions and discharge destinations by cohort.

	COS			IHS			*P for trend*	
	1995–2001	2002–2008	2009–2015	1995–2001	2002–2008	2009–2015	*COS*	*IHS*
**Imaging (%)**								
CT and/or MRI	1,379 (92.4)	1,755 (99.0)	1,186 (100)	162 (78.3)	194 (95.1)	121 (100)	*<0*.*001*	*<0*.*001*
MRI	162 (10.9)	451 (25.6)	388 (36.9)	27 (13.1)	21 (10.6)	34 (33.7)	*<0*.*001*	*<0*.*001*
**Swallow assessment**	1,414 (94.4)	1,595 (89.2)	1,072 (84.9)	202 (95.3)	176 (85.4)	97 (72.9)	*<0*.*001*	*<0*.*001*
**SU admission**	483 (32.2)	1,369 (77.1)	1.090 (86.3)	26 (12.4)	102 (50.0)	87 (65.4)	*<0*.*001*	*<0*.*001*
**>50% of stay on SU**	128 (15.2)	1,084 (68.6)	837 (85.9)	20 (11.9)	75 (38.7)	57 (58.2)	*<0*.*001*	*<0*.*001*
**Thrombolysis**	n/a	107 (10.0)	185 (17.8)	n/a	7 (5.1)	15 (13.8)	*n/a*	*n/a*
**Hospital stay**, median number of days (IQR)	19 (7–51)	13 (5–39)	7 (2–21)	21 (6–54)	20 (7–43)	17 (6–39)	*<0*.*001*	*0*.*688*
**Discharge destination**								
Home without carer	195 (30.2)	336 (24.7)	247 (24.7)	21 (29.2)	22 (19.5)	24 (27.9)	*0*.*024*	*0*.*932*
With carer / sheltered	364 (56.4)	885 (64.9)	691 (69.1)	32 (44.4)	57 (50.4)	47 (54.7)	*<0*.*001*	*0*.*203*
Nursing care	87 (13.5)	142 (10.4)	62 (6.2)	19 (26.4)	34 (30.1)	15 (17.4)	*<0*.*001*	*0*.*167*
**Care at 3 months**								
PT or OT after d/c*	75 (25.9)	187 (53.0)	323 (48.1)	3 (10.0)	15 (40.5)	30 (57.7)	*<0*.*001*	*<0*.*001*
SALT after d/c^†^	59 (14.0)	126 (25.4)	130 (37.0)	3 (7.9)	10 (22.7)	7 (41.2)	*<0*.*001*	*0*.*004*
Antihypertensives^‡^	333 (63.7)	475 (79.2)	265 (55.7)	25 (54.4)	34 (70.8)	19 (43.2)	*0*.*014*	*0*.*299*
Antiplatelets^§^	476 (75.4)	533 (76.3)	438 (74.1)	32 (56.1)	49 (73.1)	31 (68.9)	*0*.*601*	*0*.*143*
Anticoagulation**	46 (39.0)	53 (40.5)	66 (46.5)	4 (26.7)	10 (50.0)	8 (44.4)	*0*.*213*	*0*.*325*
Statin therapy^#^	32 (33.0)	323 (89.5)	285 (81.9)	0	26 (86.7)	27 (90.0)	*<0*.*001*	*0*.*003*

Data are count (%, excluding those with missing values) unless otherwise indicated, Abbreviations: PT physiotherapy, OT occupational therapy, d/c discharge, SALT speech and language therapy

* of patients with any paralysis or sensory deficit in the acute phase

† of patients with dysphasia, dysarthria, or both in the acute phase

‡ of those with hypertension

§ of those with cerebral infarction

** of those with cerebral infarction and atrial fibrillation

# of those with hypercholesterolaemia

During the study period, in-hospital mortality declined in absolute terms by 20.4% (COS) and 30.2% (IHS) respectively: following COS, in-hospital mortality was 34.1% in 1995–2001, 19.7% in 2002–2008, and 13.7% in 2009–2015, while after IHS it was 56.9% in 1995–2001, 44.3% in 2002–2008, and 26.7% in 2009–2015.

Median length of hospital stays shortened significantly for COS (19 days in 1995–2001 vs 7 days in 2009–2015, P for trend<0.001), but not IHS patients (21 days in 1995–2001 vs 17 days in 2009–2015, P for trend = 0.688). Since 1995, an increasing number of COS patients were discharged home with carer or into sheltered accommodation and less into nursing care. During that period, discharge destinations for IHS patients did not change significantly.

The proportion of eligible COS and IHS patients receiving PT, OT, or SALT in the first three months after stroke increased during the study period. IHS patients received less PT or OT than COS patients in the earlier two cohorts, but not the latest cohort. However, most differences were statistically non-significant. There were no significant differences between COS and IHS in secondary prevention therapies at three months post-stroke, apart from IHS patients having had lower rates of antiplatelet therapy at three months in the earliest cohort (P = 0.002).

Multivariable logistic regression showed that, after adjusting for socio-demography, comorbidities, case mix variables, and stroke subtype, IHS was independently associated with lower rates of SU admission in each cohort (1995–2001: OR 0.49, 95%CI 0.29–0.82, 2002–2008: 0.29, 0.18–0.45, 2009–2015: 0.22, 0.11–0.43). Including only ischaemic strokes after 2003 and using the same adjustment variables as above, IHS was also linked to lower rates of thrombolysis, but this did not reach statistical significance (OR 0.67, 0.37–1.19). Including the whole study population, IHS was independently associated with higher rates of in-hospital mortality after adjusting for the variables above (OR 1.60, 1.16–2.21), but this association was not statistically significant in the latter two cohorts (1995–2001: OR 1.33, 0.79–2.22, 2002–2008: 1.95, 1.19–3.19, 2009–2015: 1.63, 0.61–4.34). Similarly, IHS was significantly associated with higher rates of discharge into nursing care overall (OR 1.77, 1.17–2.67), but after stratification this association only remained significant for the 2002–2008 cohort (1995–2001: OR 1.56, 0.79–3.08, 2002–2008: 1.95, 1.05–3.62, 2009–2015: 1.69, 0.51–5.60).

Fig 1 presents three-month and five-year outcomes as death, severe/moderate disability, and mild/no disability stratified by cohort. There were significant improvements of outcomes in both groups, but mild/no disability was less common in IHS patients than COS patients in each cohort.

**Fig 2:**
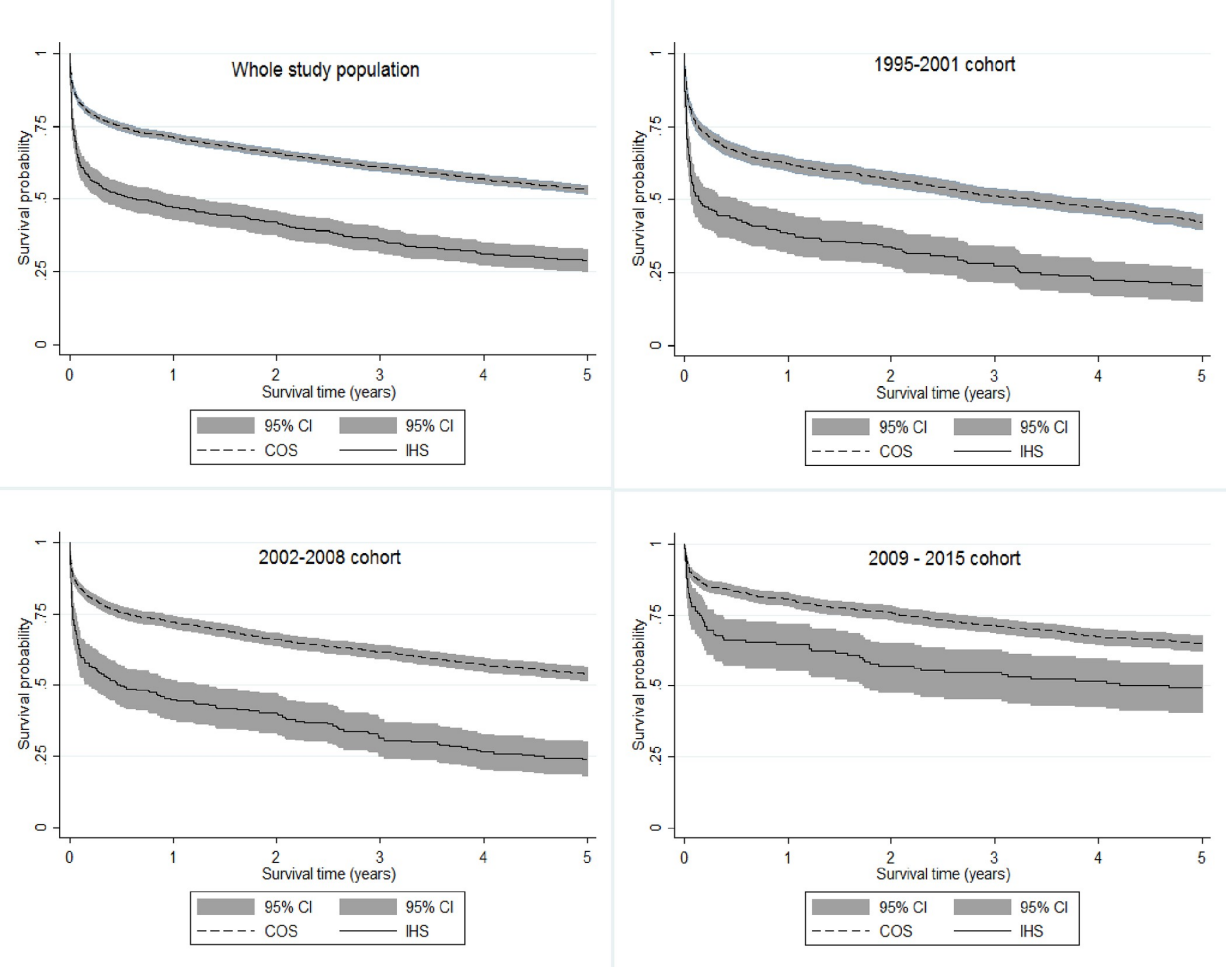
Kaplan-Meier survival estimates over 5 years following stroke.

Fig 2 shows unadjusted survival curves over the five years following index stroke stratified into COS and IHS for the overall study population and for each cohort. Although survival rates improved in both groups (COS and IHS: log rank test between cohorts: P<0.001), survival rates were significantly lower in IHS compared to COS patients in each cohort (each cohort: log rank test between COS and IHS: P<0.001). In all cohorts, most of the divergence between COS and IHS patients’ survival occurred in the early phase post-stroke.

After adjusting for socio-demography, comorbidities, case mix, stroke subtype, and hospital care processes, IHS remained associated with higher mortality rates in each cohort, while SU admission was consistently associated with lower mortality rates (Table 3).

**Table 3:** Factors influencing all-cause mortality over 5 years post-stroke, HR (95%CI)*.

	1995–2001	2002–2008	2009–2015	1995–2015
**IHS, vs COS**	1.27 (1.03–1.57)	1.24 (0.99–1.55)	1.39 (0.95–2.04)	**1.24 (1.08–1.43)**
**Imaging, vs none**	0.26 (0.18–0.40)	0.26 (0.06–1.09)	N/A	**0.33 (0.23–0.47)**
**SU admission, vs none**	0.71 (0.60–0.84)	0.56 (0.47–0.68)	0.60 (0.40–0.90)	**0.65 (0.58–0.73)**
**Age, vs <65**				
**65–74**	1.89 (1.50–2.38)	3.15 (2.43–4.09)	3.54 (2.26–5.55)	**2.51 (2.14–2.94)**
**≥75**	3.24 (2.61–4.04)	5.50 (4.30–7.03)	7.59(5.09–11.33)	**4.53 (3.91–5.26)**
**Female, vs male**	1.02 (0.88–1.19)	0.94 (0.81–1.09)	0.92 (0.72–1.18)	**0.99 (0.90–1.09)**
**Ethnicity, vs white**				
**Black**	0.82 (0.66–1.01)	0.89 (0.72–1.09)	0.79 (0.60–1.05)	**0.85 (0.75–0.96)**
**other**	0.53 (0.35–0.80)	0.73 (0.51–1.05)	0.66 (0.38–1.14)	**0.63 (0.49–0.80)**
**Stroke subtype, vs IS**				
**PICH**	0.90 (0.73–1.10)	1.18 (0.95–1.48)	0.71 (0.46–1.10)	**0.97 (0.85–1.12)**
**SAH**	0.92 (0.63–1.36)	0.71 (0.44–1.15)	0.24 (0.03–1.72)	**0.63–1.13)**

*adjusted for the variables shown, as well as socio-economic status, pre-stroke vascular risk factors, and case mix variables, Abbreviations: IS ischaemic stroke, PICH primary intracerebral haemorrhage, SAH subarachnoid haemorrhage

## Discussion

This is the first study comparing temporal trends of stroke care and outcomes between IHS and COS patients. Previous studies have compared both groups of patients regarding care and short-term outcomes, mostly up to three months[2, 5–9]. However, they did not investigate long-term outcomes nor, importantly, if the improvements in care and outcomes reported for stroke patients generally were also experienced by those having IHS. This is particularly relevant, as during the study period from 1995 to 2015 guidelines and service structures of stroke care have changed significantly. In 2010, the study area within London saw the centralisation of stroke services, during which a relatively large number of local hospitals admitting acute stroke patients was reduced to a small number of hyper-acute stroke units[22].

Analysing the large cohort of stroke patients included in the SLSR, which is continuously ongoing since 1995, allowed us to investigate changes in care and outcomes over the last 20 years. The main findings from this study demonstrate that care and outcomes of IHS have improved significantly during that time. However, rates of evidence-based, acute care interventions are still lower and outcomes poorer than those of COS, even after adjusting for differences in socio-demography, comorbidities, and case mix between the two groups.

In this study, the proportion of strokes which occurred in hospital (10.8%) was significantly higher than the 5.7% reported in the national audit of hospital-admitted stroke patients[1]. This probably reflects the high-risk patient group treated in the two main hospitals of the study area, which both serve as major tertiary centres for various specialities leading to a higher risk of IHS[5], especially cardiothoracic surgery, interventional cardiology, and neurosurgery.

### Processes of care

Measures of acute care (imaging, SU admission, and thrombolysis rates) and early-phase therapies (secondary prevention, PT, OT, and SALT) show increased rates in both groups. This reflects the implementation of evidence-based stroke care guidelines[23] across both groups of stroke patients. Imaging rates of 100% and rates of SU admissions of well over 80% and 60% for COS and IHS respectively indicate major improvements in clinical practice over the study period and are also similar to current national estimates[1]. Despite these positive trends, this study shows a continuation of the previously reported disadvantage of IHS patients[2, 9]: dedicated, multi-disciplinary SU care and thrombolysis remained significantly less common after IHS than COS in the most recent cohort.

There are valid medical reasons for not being transferred to a SU, such as serious competing illnesses which warrant primary treatment in critical care, or post-surgical or coronary care units. This study does not contain data on the initial admission diagnosis or hospital procedures of IHS patients and therefore the causes for not being transferred to a SU could not be investigated in detail. However, when analysing the association between IHS and rates of SU admission, we adjusted for as many of the validated case mix variables as possible[24], including comorbidity profiles and clinical assessments at the time of maximal impairments. Within the limitation of this analysis, a significant, independent link between IHS and lower rates of SU admission was confirmed in this multivariable analysis.

Avoidable, non-medical barriers of entering the standard (usually admission-) stroke care pathway, as found in previous studies[2, 25], might continue to play an additional role, e.g. physicians and nurses managing IHS patients being less familiar with stroke and the evidence-based interventions required compared to staff routinely managing stroke admissions. This can lead to delays in symptom recognition and diagnosis, a lower priority of transfers to SUs from wards other than the emergency department, and generally less adherence to consensus quality process measures of care[26].

In previous reports[2, 9, 27, 28], thrombolysis rates after IHS varied widely from 3.7 to 15.7%, presumably due to the different time points of data collection, different denominators used, and the generally low numbers involved. The thrombolysis rate for ischaemic IHS found here for the 2009–2015 cohort (13.8%) fits into the previously reported range and nearly reaches a 2006 estimate, that up to 14% of IHS patients could potentially be eligible for thrombolysis[29].

However, compared to the corresponding rates for COS, IHS thrombolysis rates were in absolute terms 4.9% and 4.0% lower in 2002–2008 and 2009–15 respectively. This dataset does not include information on contraindications to thrombolysis, but previous reports, which also found lower thrombolysis rates after IHS[3, 9], pointed to higher rates of clinical contraindications in IHS patients, such as recent major surgery or severe medical disorders[4, 26, 27, 30]. Additionally, they found lower thrombolysis rates also caused by significant and often preventable delays: delayed symptom recognition, e.g. due to sedation or less experienced hospital staff[3, 26], less emergency stroke specialist referrals[4, 30, 31], and longer times from stroke onset to imaging or treatment[25, 32]. This combination of higher rates of contraindications to thrombolysis, as well as preventable delays in symptom recognition and diagnosis are plausible explanation for the lower thrombolysis rates found here.

Our cohort pre-dates the introduction of thrombectomy services in the study area, but thrombectomy is now available and recommended for certain patients with ischaemic stroke, including those with contraindications to thrombolysis [23]. As a significant number of IHS patients have been shown to have such contraindications, thrombectomy might offer an effective, alternative treatment particularly in this patient group. The increasing availability of this treatment option reinforces the importance of early diagnosis in IHS patients.

In contrast to the increasing rates of brain imaging, thrombolysis, and SU admission, the frequency of swallowing assessments decreased in both groups. This has been reported before[33], but differs from results of the national stroke audit SSNAP, which reports rising rates of swallowing assessment, reaching 82.6%[1]. It is unclear if this is a true decline, possibly due to the increasing number of people with milder strokes being assessed less consistently, or an artefact due to worsening documentation[33]. In either case, it does suggest shortcomings in care.

This study showed significant improvements in the rate of early rehabilitation and secondary prevention therapies post-discharge after both COS and IHS, with no significant differences between the two groups. This encouraging finding suggests, that IHS patients receive similar levels of care after the acute phase. It might reflect a growing familiarity with evidence-based stroke care guidelines among medical staff, especially outside dedicated stroke wards or the acute hospital setting.

### Outcomes

This study found improved outcomes for both groups of patients, probably linked to the observed decline in stroke severity and increased use of evidence-based treatment in both groups. Nevertheless, outcomes after IHS remained significantly poorer than after COS: longer hospital stays, higher rates of in-hospital and long-term mortality, and less favourable functional outcomes in the short- and long-term, leading to higher rates of nursing home care. Additionally, Fig 2 demonstrates that most of the difference in overall survival rates following IHS is due to lower survival rates in the early phase post-stroke in this patient group, underlining the relevance of acute complications and timely interventions in this patient group.

The lower rates of process measures of evidence-based care following IHS discussed above, such as SU admissions and thrombolysis, might contribute to some of the generally poorer outcomes after IHS, as supported by the significant, independent association between dedicated, multidisciplinary SU care and improved survival rates observed in this study.

Poorer outcomes after IHS might be expected even with optimal care, since this patient group is older, with more comorbidities and probably more contraindications to stroke treatment. Associations between IHS and outcomes were therefore adjusted for the case mix variables suggested by Davenport et al.[24] and the validated case mix variables that have been shown to predict poor outcomes and are sufficient in quality and precision. These included socio-demography, pre-stroke disability, comorbidities, stroke subtype and clinical impairments in the acute phase. As IHS remained linked to poorer outcomes after adjustment, those factors did not fully explain the differences in outcomes between COS and IHS.

From out study alone, we cannot determine the extent that lower survival was caused by worse care, or by baseline differences in IHS and COS patients. Further confounding factors, which are likely to play a role, but were unavailable and therefore unadjusted for, include the initial admission diagnosis of IHS patients and markers of patient frailty, as well as factors relating to hospital care processes, such as the timeliness, quality, and intensity of treatment. Those latter factors have been shown to be significantly poorer for IHS patients, as reported in previous studies[26, 32] and discussed above.

### Further methodological considerations

This observational cohort study has strengths and limitations. In addition to the weaknesses discussed above, particularly the lack of information on the initial admission diagnosis of IHS patients, this study had a loss to follow-up rate of around 20% after accounting for deaths[16], which may introduce bias. However, estimates from analyses of patients with complete data did not differ significantly from those presented. Inner-city populations with many migrant families are relatively mobile, making long-term follow-ups challenging. Significant efforts were made to update patient contact details via hospital or GP records, and family members.

On the other hand, this study has several strengths. As a register which is continuously ongoing since 1995, the SLSR provides a large, multi-ethnic cohort of over 5000 admitted stroke patients, including more than 550 IHS patients, one of the largest samples of IHS patients analysed so far. Additionally, the dataset is very unusual for covering more than 20 years. The large sample allows stratification into multiple groups, while still providing significant numbers and statistical power to detect differences and trends over time. Its long-term follow-ups produce data for the analysis of long-term functional outcomes. Additionally, as many validated process and outcome measures have been collected continuously since 1995, during which time policies and service structures have changed significantly, their level of implementation and effects on outcomes could be evaluated. At the same time, variables which were not available for the earliest cohort, such as the TOAST classification of stroke[34] and the NIHSS score[17] as a measure of stroke severity, could not be included in the whole analysis.

### Conclusions

Patients who experience a stroke while in hospital have the potential advantage of ready access to high quality stroke care. This study demonstrates an increase in the use of evidence-based treatments and improvement in outcomes after IHS similar to those after COS, but also a continuing inequality in the provision of evidence-based acute care between both groups. Previous research has shown that a significant part of this inequality is due to valid clinical reasons but also avoidable shortcomings and delays, which are possibly still playing a role. This might imply some room for further improvements.

In order to further advance the care of IHS patients, all hospitals require algorithms and protocols specifically tailored to IHS patients[23], with clear lines of communication and allocation of responsibilities (e.g. in-hospital stroke teams). They need to be supported by staff training programs and audits to ensure systematic implementation. Raising awareness of the urgency of diagnosis and treatment is particularly important in high-risk clinical areas such as cardiology, renal wards, or cardiothoracic units, but also generally across all hospital areas. Efforts towards these objectives are being undertaken, such as specifically discussing IHS in the 2016 National Stroke Guidelines[23] or multidisciplinary-team education on some high-risk hospital wards. Future studies should investigate the causes for delays in symptom recognition and referral, as well as persisting obstacles to SU transfer, justified or otherwise. This will help clinicians and service leads to develop stroke services in a way that meets IHS patients’ specific needs by identifying critical parts of the hospital pathway.
